# Pre-Transplant Platelet Refractoriness and Alternative Donors Are Associated With Cytomegalovirus Retinitis in Hematopoietic Stem Cell Transplantation for Severe Aplastic Anemia

**DOI:** 10.3389/fcimb.2022.870296

**Published:** 2022-03-15

**Authors:** Yuehong Zhang, Yuqin Liang, Xu Zhang, Shunqing Wang, Jinpeng Cao, Zongyin Gao, Ling Li, Wenjian Mo

**Affiliations:** ^1^Department of Ophthalmology, Guangzhou First People’s Hospital, School of Medicine, South China University of Technology, Guangzhou, China; ^2^Department of Hematology, Guangzhou First People’s Hospital, School of Medicine, South China University of Technology, Guangzhou, China; ^3^State Key Laboratory of Respiratory Disease & National Clinical Research Center for Respiratory Disease, Guangzhou Institute of Respiratory Health, First Affiliated Hospital of Guangzhou Medical University, Guangzhou Medical University, Guangzhou, China; ^4^Guangzhou Laboratory, Bio-Island, Guangzhou, China; ^5^Department of Biology, University of North Dakota, Grand Forks, ND, United States

**Keywords:** ocular infection, cytomegalovirus retinitis, hematopoietic stem cell transplantation, severe aplastic anemia, risk factor

## Abstract

**Background:**

Cytomegalovirus retinitis is a severe, vision-threatening opportunistic infection in an immunodeficient population. Reports on cytomegalovirus retinitis in hematopoietic stem cell transplant recipients due to severe aplastic anemia have been scant. This study assessed the risk of cytomegalovirus retinitis in relation to the pre-transplant status of severe aplastic anemia patients.

**Methods:**

We conducted a retrospective nested case-control study of cytomegalovirus retinitis among severe aplastic anemia patients receiving allogeneic hematopoietic stem cell transplants in a tertiary care institution that attends severe aplastic anemia patients from southern China from January 1, 2013 to December 31, 2018. Each cytomegalovirus retinitis case was matched with four controls without cytomegalovirus retinitis by age and gender. Thirteen pre-transplant parameters were chosen to compare the risk factor levels between the cases and controls. Multivariable logistic regressions were used to estimate the odds ratios (ORs) and 95% confidence intervals (CIs).

**Results:**

A total of 361 severe aplastic anemia patients received hematopoietic stem cell transplants in the study period 2013–2018 in our medical institution, and 31 (8.58%) developed cytomegalovirus retinitis. Cytomegalovirus retinitis was diagnosed in the median of 148 days after transplantation. We confirmed platelet refractoriness more frequently in cases than in controls (*p* = 0.0005). Compared with human leukocyte antigen-matched sibling donors, alternative donors were significantly more prone to cytomegalovirus retinitis (*p* = 0.0009). After stepwise selection in multivariate logistic regression, platelet refractoriness (OR 5.41, 95% CI 1.98–15.39), haploidentical donor (OR 7.46, 95% CI 2.19–34.87), and unrelated donor (OR 8.38, 95% CI 2.30–41.34) were associated with an increased risk of cytomegalovirus retinitis.

**Conclusions:**

Pre-transplant platelet refractoriness and alternative donors were significant predictors of cytomegalovirus retinitis in severe aplastic anemia recipients. These results highlight the importance of accounting for existing risks while developing prevention strategies and preemptive treatment for severe aplastic anemia recipients. We recommend that the platelet count be closely monitored and thrombopoietin be properly applied during the period when cytomegalovirus retinitis is prone to occur.

## Introduction

Cytomegalovirus (CMV) is a double-stranded DNA virus of the herpes family. CMV infection can be a primary infection, reinfection, or reactivation. The clinical manifestations and outcome of CMV infection are closely related to the immune status of individuals, ranging from mild asymptomatic infection to severe organ damage and even death. CMV infection has a high morbidity and mortality in patients with severe immune deficiency, which can induce invasive disease of one or more organs such as brain, gastrointestinal tract, lung, liver and retina (Ljungman et al., 2017). CMV retinitis (CMVR), a progressive retinal necrotizing disease, is an ocular opportunistic infection in patients who cannot produce effective immune response to CMV, which mainly occurs in patients with human immunodeficiency virus infection, immunosuppressive therapy and organ transplant (Munro et al., 2019; Stern et al., 2019).

Severe aplastic anemia (SAA) is a progressive autoimmune disease characterized by peripheral pancytopenia and marrow hypoplasia, which can be treated effectively with allogeneic hematopoietic stem cell transplantation (HSCT) (Bacigalupo, 2017; Kumar et al., 2017). The rapid advances in HSCT techniques in recent decades have broadened the indication of HSCT in SAA recipients. The donor type has varied from initial human leucocyte antigen (HLA)-matched sibling donor (MSD) and then extended to HLA-haploidentical donor (HID) or HLA-matched unrelated donor (URD) (Georges and Storb, 2016; Xu et al., 2017). The transplant conditioning regimen always contains antithymocyte globulin (ATG) for SAA, which is unique to malignant hematological diseases, and it is generally accepted that immunosuppressive therapy with cyclosporine A (CSA) after transplantation lasted for more than one year to avoid graft failure and graft-versus-host disease (George et al., 2013; Li et al., 2018). CMVR is a common late-onset HSCT-related ocular complication that has the potential to cause severe visual impairment and blindness (Crippa et al., 2001; Hiwarkar et al., 2014; Lu et al., 2016). In recent years, many studies on CMVR in non-human immunodeficiency virus individuals have focused on HSCT recipients with malignant hematological diseases (Ando et al., 2020). However, HSCT-related CMVR has rarely been mentioned in patients with SAA. For SAA patients undergoing HSCT, it remains unclear whether some unique pre-transplant clinical features of the disease may predispose them to post-transplant CMVR.

The known risk factors for CMVR in HSCT recipients include donor and recipient serostatus, high-dose corticosteroids, T-cell depletion, graft-versus-host disease, a long duration of CMV viremia, repeated treatment with ATG, and transplantation from the alternative donor (Ljungman et al., 2011; Jeon et al., 2012; Camargo and Komanduri, 2017). To date, there has been very limited published research investigating pre-transplant risk factors predicting post-transplant CMVR in SAA HSCT recipients. This study was designed to identify potential SAA HSCT recipients who are at increased risk for post-transplant CMVR by comparing pre-transplant parameters between patients with CMVR and non-CMVR controls. It is helpful to reduce the risk of CMVR so that SAA patients benefit from optimization of preemptive therapy with antiviral prophylaxis.

## Methods

### Patient Selection

A retrospective, matched, case-control study of HSCT recipients with SAA was conducted. The subjects were patients who underwent allogeneic HSCT at Guangzhou First People’s Hospital in China from January 1, 2013 to December 31, 2018 due to SAA. Cases were CMVR patients who were diagnosed within 2 years after HSCT. Controls were those who did not develop CMVR at least 2 years after transplantation, excluding those who had no complete clinical data due to the missing follow-up or deaths, had no regular fundus examination records, or were diagnosed with acute retinal necroses. Collected data from an electronic patient record included demographic data, clinical findings, and virology records. This study was approved by the institutional review board of Guangzhou First People’s Hospital (IRB K-2021-121-01) and was conducted in accordance with the Helsinki Declaration.

### Ascertainment of Cases and Controls

All patients who met the discharge diagnosis on the first page of the medical record system of Guangzhou First people’s Hospital and met the conditions of SAA (disease Code: D61.905) and HSCT (disease Code: Z94.800) were identified. CMVR was diagnosed according to typical ophthalmological signs judged by an experienced ophthalmologist using fundus pre-set lens with fully dilated pupils. For atypical presentation, the diagnosis of CMVR was supported by CMV load documented in aqueous humor by real-time quantitative polymerase chain reaction (PCR) analysis (Ljungman et al., 2017). There are three main fundus presentations of CMVR. The granular form is characterized by whitish granular lesions, often starting at the retinal periphery. The hemorrhagic/edematous form is more aggressive, which can rapidly lead to extensive yellowish foci of retinal necrosis intermingled with retinal hemorrhages. The frosted branch angiitis form is manifested by an occlusive retinal vasculitis with exuberant whitening of the vessel wall, as if it were frozen (Vadlapudi et al., 2012). Non-CMVR controls were matched to CMVR cases by a ratio of 4:1 using frequency matching on age and gender. The following age subgroups were used for matching: 15–25, 26–35, 36–45, and 46–50 years. A list of numbers computer-generated by a research member (Jinpeng Cao) was used to match the controls.

### The Measurement of CMV Load

All patients who received HSCT in our medical institution were routinely monitored the viruses DNA levels including CMV and Epstein–Barr virus (EBV) in the peripheral venous blood using real-time PCR before HSCT and then once weekly for at least three months. It was defined as CMV or EBV viremia when 2 consecutive levels of CMV-DNA > 500 copies/mL or EBV-DNA > 1000 copies/mL were detected in peripheral blood. The CMV load data was reported in copies/mL. Anterior aqueous humor for CMV-DNA PCR testing was obtained through paracentesis in patients diagnosed with CMVR or who were highly suspected to have CMVR through an ophthalmologic examination.

### Exposures and Definition

Thirteen pre-transplant parameters were chosen to compare the risk factor levels between the cases and controls. We considered the diagnosis, interval from diagnosis to HSCT, red blood cell infusion, platelet infusion, immunotherapy failure, platelet refractoriness, ferritin amount, uncontrolled infection, donor type, donor age, blood types of donor to recipient, gender matching of donor/recipient and stem cell source. SAA is defined by at least two of the following: reticulocytes < 50 × 10^9^/L or 20 × 10^9^/L by manual counting, platelet count < 20 × 10^9^/L, and neutrophil count < 0.5 × 10^9^/L. In very SAA (vSAA), neutrophil counts are < 0.2 × 10^9^/L (Passweg and Marsh, 2010). Immunotherapy failure is defined as the failure to respond to the best available immunosuppressive treatment lasting for 6 months, including CSA therapy or combined ATG and CSA therapy (Deeg et al., 2006). Platelet refractoriness is defined as lower-than-expected post-transfusion count increments after repeated platelet transfusions (Cohn, 2022). The donor type included MSD, URD and HID, the latter two of which describe alternative donors.

### Statistical Analyses

We evaluated the association between pre-transplant parameters and CMVR as the primary outcome. Continuous data are presented as mean and standard deviation, whereas categorical data are presented as percentages. T-test was used for continuous variables, and chi-square test was used for categorical variables. Univariate and multivariate logistic regression analyses were used to explore the effect factor for CMVR and demographic or clinical variables. Odds ratio (OR) and corresponding 95% confidence intervals (95% CI) were presented as effect estimates. The multivariate model uses stepwise selection with an entry level of 0.1, and a variable remains if it meets the 0.05 level. A bootstrap resampling method using 100 bootstrap samples was used to internally validate the results. Each time, a bootstrap was drawn from the original dataset and the correspondent *p* value was calculated. The number of times with a *p* value less than 0.05 was counted. The model performance was evaluated using the area under the receiver operating characteristic (ROC) curve (AUC). We used R software (Version 4.0.4) to analyze the data. *P* values less than 0.05 were considered statistically significant.

## Results

### Patient Characteristics

**Figure 1:**
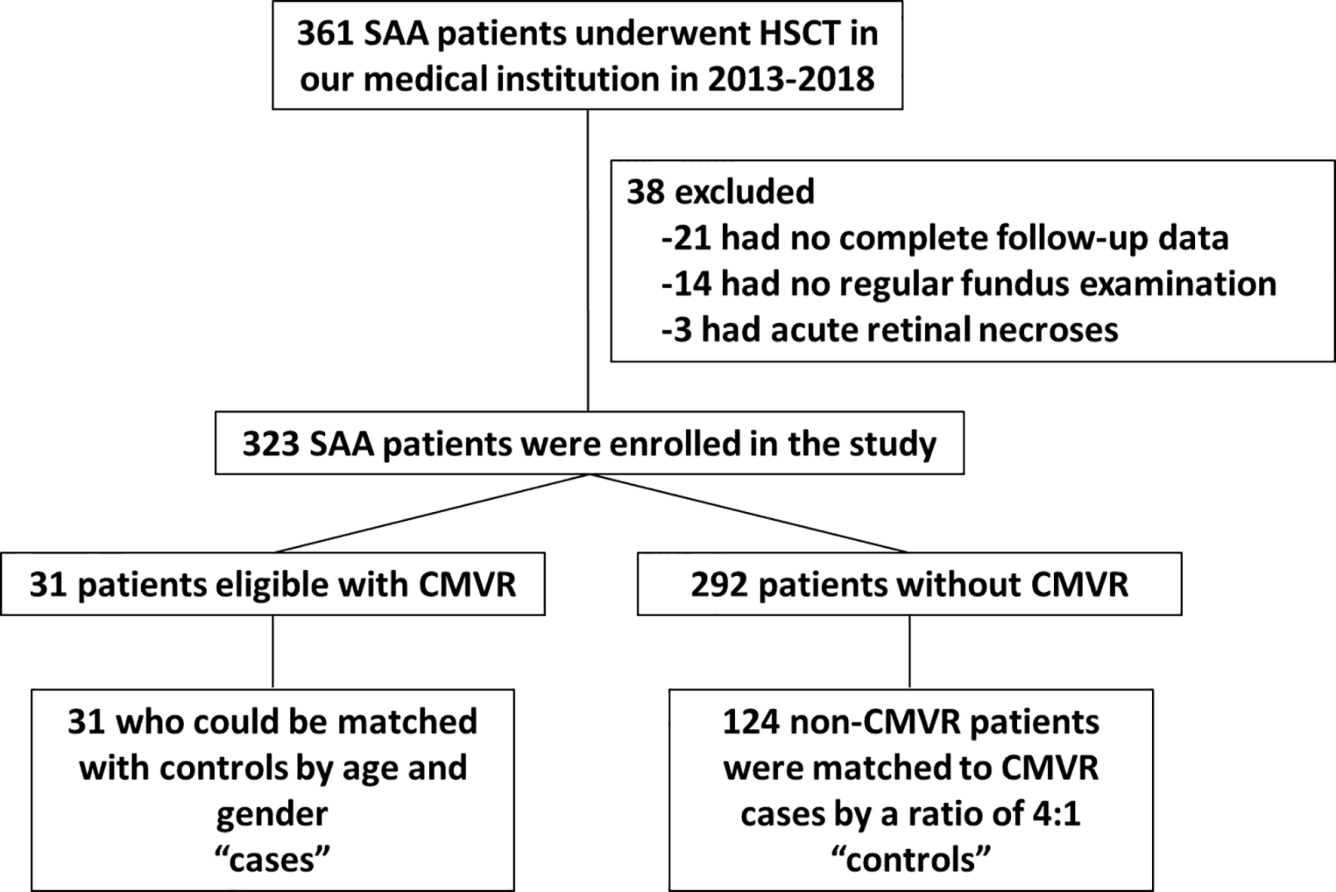
Flow chart of SAA patients selected for the study. SAA, severe aplastic anemia; HSCT, hematopoietic stem cell transplantation; CMVR, cytomegalovirus retinitis.

There were 361 SAA patients who received HSCT in the study period 2013–2018 in our medical institution. Twenty-one patients were excluded because they had no complete follow-up data. Fourteen patients were excluded because they had no regular fundus examination records. Three patients were excluded due to acute retinal necroses. A total of 31 CMVR patients and 292 non-CMVR patients were eligible for this study. According to our design of nested case-control study, we finally included 31 cases and 124 age- and gender-matched controls (4 times number of cases) in this study (**Figure 1**). The average age of the cases was 30.6 ± 9.3 years (range, 15–50 years), and it was 28.8 ± 9.1 years (range, 15–49 years) in the controls; 64.5% of the cohort was male. All 31 CMVR cases developed CMV viremia before the diagnosis of CMVR. The mean time of onset of CMV viremia was 31.84 ± 14.59 days, and the duration was 55.72 ± 66.38 days. The average time to reach the peak level of CMV DNA in the blood after transplantation was 50.60 ± 25.36 days, and the corresponding CMV load was 9.77 × 10^3^ ± 1.27×10^4^ copies/mL. The median time to CMVR diagnosis was 148 days (range, 87–386 days) post-transplant. The median level of CMV DNA in aqueous humor was 8745 copies/mL (range, 534–1.8 × 10^6^ copies/mL) by quantitative PCR. The characteristics of the patients are summarized in **Table 1**. **Table 2** compared the 13 pre-transplant parameters between two groups. The cases and controls did not differ with respect to the diagnosis, interval from diagnosis to HSCT, red blood cell infusion, platelet infusion, immunotherapy failure, ferritin amount, uncontrolled infection, donor age, blood types of donor to recipient, gender matching of donor and recipient, or stem cell source. Pre-transplant platelet refractoriness was more frequently seen in CMVR cases than in non-CMVR controls (*p* = 0.0005). The proportion of platelet refractoriness in the cases was 35.5%, whereas it was 10.5% in the controls. Regarding the donor type, CMVR cases were associated with a higher proportion of alternative donors (90.3%) than non-CMVR controls (45.2%) (*p* = 0.0009).

**Table 1:** Basic characteristics of 155 patients included in this study.

General information	Total	Cases	Controls
Recipient number	155	31	124
Recipient age-yr	29.14 ± 9.12	30.6 ± 9.3	28.8 ± 9.1
Recipient age-no.			
< 25 yr	65 (41.9%)	13	52
26–35 yr	55 (35.5%)	11	44
36–45 yr	25 (16.1%)	5	20
46–50 yr	10 (6.5%)	2	8
Recipient gender-no.(%)			
Male	100 (64.5%)	20	80
Female	55 (35.5%)	11	44
Donor age-yr	31.03 ± 11.67	30.6 ± 12.9	31.1 ± 11.4
Donor age-no.(%)			
< 25 yr	57 (36.8%)	14	43
26–35 yr	52 (33.5%)	10	42
36–45 yr	24 (15.5%)	1	23
46–64 yr	22 (14.2%)	6	16
Donor gender-no.(%)			
Male	96 (61.9%)	22	74
Female	59 (38.1%)	9	50

**Table 2:** Comparison of 13 pre-transplant parameters between CMVR cases and age- and gender-matched controls.

Variables		Cases (n=31)	Controls (n=124)	*p* value
Diagnosis-no.(%)				
SAA		18 (58.1%)	89 (71.8%)	0.1331^#^
vSAA		13 (41.9%)	35 (28.2%)	
Interval from diagnosis to HSCT-mth		42.1 ± 63.2	26.7 ± 49.3	0.2701^^∧^^
Interval from diagnosis to HSCT-no.(%)				
< 1 mth		9 (29.0%)	48 (38.7%)	0.5469^#^
1–12 mth		11 (35.5%)	42 (33.9%)	
> 12 mth		11 (35.5%)	34 (27.4%)	
Red blood cell infusion-no.(%)				
< 20U		12 (38.7%)	73 (58.9%)	0.1088^#^
> 20U		19 (61.3%)	51 (41.1%)	
Platelet infusion-no.(%)				
< 10U		12 (38.7%)	66 (53.2%)	0.3025^#^
> 10U		19 (61.3%)	58 (46.8%)	
Immunotherapy failure-no.(%)				
No		15 (48.4%)	76 (61.3%)	0.3651^#^
Yes		16 (51.6%)	48 (38.7%)	
Platelet refractoriness-no.(%)				
No		20 (64.5%)	111 (89.5%)	0.0005^#^
Yes		11 (35.5%)	13 (10.5%)	
Ferritin amount		1513.9 ± 918.3	1401.5 ± 256.03	0.9836^∧^
Uncontrolled infection-no.(%)				
No		28 (90.3%)	112 (90.3%)	1.0000^#^
Yes		3 (9.7%)	12 (9.7%)	
Donor type-no.(%)				
MSD		3 (9.7%)	68 (54.8%)	0.0009^#^
Alternative donor (HID and URD)		28 (90.3%)	56 (45.2%)	
Donor age-yr		30.6 ± 12.9	31.1 ± 11.4	0.7923^∧^
Donor age-no.(%)				
< 25 yr		14 (45.2%)	43 (34.7%)	0.1387^#^
26–35 yr		10 (32.3%)	42 (33.9%)	
36–45 yr		1 (3.2%)	23 (18.5%)	
46–64 yr		6 (19.3%)	16 (12.9%)	
Blood types of donor to recipient-no.(%)				
Match		18 (58.1%)	58 (46.8%)	0.4742^#^
Mismatch		13 (41.9%)	66 (53.2%)	
Donor-recipient gender match-no.(%)				
Male for male		14 (45.2%)	44 (35.5%)	0.6470^#^
Female for female		3 (9.7%)	14 (11.3%)	
Male for female		8 (25.8%)	30 (24.2%)	
Female for male		6 (19.3%)	36 (29.0%)	
Stem cell source-no.(%)				
BM + PB		20 (64.5%)	92 (74.2%)	0.2641^#^
PB		11 (35.5%)	32 (25.8%)

CMVR, cytomegalovirus retinitis; HSCT, hematopoietic stem cell transplantation; HID, haploidentical donor; URD, unrelated donor; BM, bone morrow; PB, peripheral blood. ^∧^T-test; ^#^chi-square.

### Univariate Analysis

We further explored the potential effect factors for CMVR using univariate logistic regression analysis. As shown in **Table 3**, pre-transplant platelet refractoriness, alternative donors, and vSAA were associated with an increased risk of developing CMVR. Pre- transplant platelet refractoriness (*p* = 0.0005, OR 5.17, 95% CI 2.06–13.08) had an increased risk of CMVR. Compared with MSD, alternative donations (HID and URD) were significantly more associated with CMVR cases (*p* = 0.0011, OR 8.58, 95% CI 2.65–38.59; *p* = 0.0044, OR 6.99, 95% CI 2.05–32.42, respectively). Further, vSAA (*p* = 0.0895, OR 2.00, 95% CI 0.89–4.45) was also associated with an increased risk of developing CMVR compared with SAA.

**Table 3:** Univariate logistic regression analysis of risk factors for CMVR.

Variables		Univariate		
		*p* value	Odds Ratio	95%CI
Diagnosis				
SAA			ref	
vSAA		0.0895	2.00	0.89–4.45
Platelet refractoriness				
No			ref	
Yes		0.0005	5.17	2.06–13.08
Donor type				
MSD			ref	
HID		0.0011	8.58	2.65–38.59
URD		0.0044	6.99	2.05–32.42
Trend		0.0039		

CMVR, cytomegalovirus retinitis; SAA, severe aplastic anemia; vSAA, very severe aplastic anemia; MSD, human leukocyte antigen-matched sibling donor; HID, haploidentical donor; URD, unrelated donor.

### Stepwise Selection and Final Model Construction

**Figure 2:**
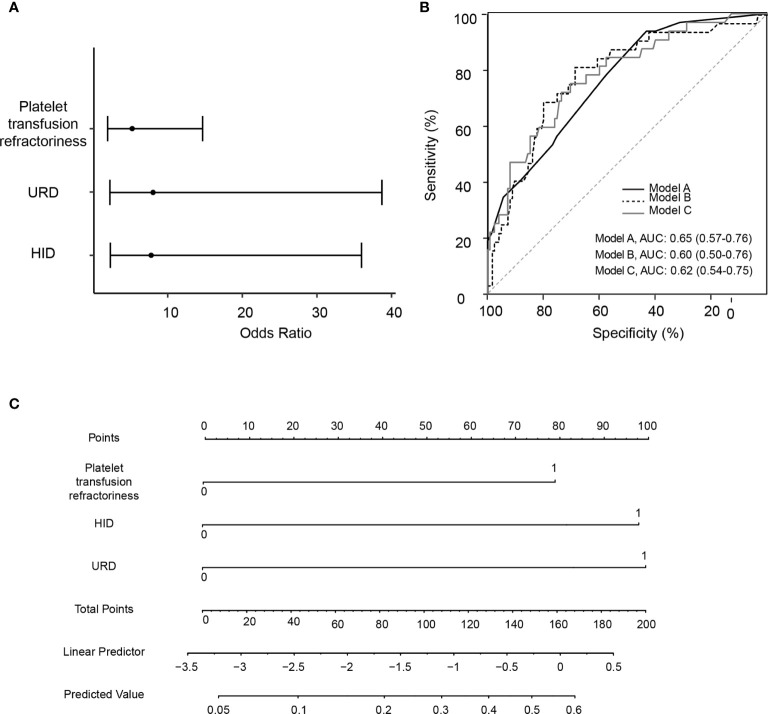
Construction and validation for the logistic regression models for pre-transplant variables and risk of cytomegalovirus retinitis. **(A)** A forest plot of odds ratio values with 95% confidence intervals in logistic regression model A. **(B)** Area under the receiver operating characteristic curve (AUC) for Model A, Model B (adjusted by age and gender), and Model C (adjusted by age, gender and diagnosis). **(C)** The nomogram for evaluating the risk for cytomegalovirus retinitis, with the rows ranging from 2 to 4 representing the included variables. The risk for cytomegalovirus retinitis among severe aplastic anemia recipients was estimated by plotting on each variable axis. A vertical line was drawn from that value to the top points scale to determine the number of points that were assigned by that variable value. The points from each variable value were then summed. The sum on the total points scale was located and vertically projected onto the bottom axis, and then the predicted value of risk for cytomegalovirus retinitis was obtained. URD, unrelated donor; HID, haploidentical donor; AUC, area under the receiver operating characteristic curve.

In the multivariate logistic regression, the stepwise model selection method was used with selected potential, and two variables were included in the final model (**Table 4** and **Figure 2A**). Pre-transplant platelet refractoriness (*p* = 0.0011, OR 5.41, 95% CI 1.98–15.39), HID (*p* = 0.0034, OR 7.46, 95% CI 2.19–34.87), and URD (*p* = 0.0030, OR 8.38, 95% CI 2.30–41.34) were associated with an increased risk of developing CMVR. To explore the effects of demographic and basic clinical variables, the other two models were set up by adjusting gender, age, and diagnosis. The risk factors were stable in all models. In model B, platelet refractoriness (*p* = 0.0006, OR 7.09, 95% CI 2.36–22.86), HID (*p* = 0.0035, OR 7.32, 95% CI 2.15–34.02), and URD (*p* = 0.0025, OR 8.61, 95% CI 2.38–41.88) were risk factors for CMVR. In model C, platelet refractoriness (*p* = 0.0005, OR 7.78, 95% CI 2.54–25.89), HID (*p* = 0.0051, OR 6.90, 95% CI 2.00–32.51), and URD (*p* = 0.0021, OR 9.33, 95% CI 2.52–46.71) were risk factors for post-transplant CMVR in SAA HSCT recipients. We stratified the main effects analysis by different host characteristics (**Table 5**). Univariate analyses of both strata for all other variables showed significant associations between the risk factors and CMVR. In multivariate analyses, the significant association was stable.

**Table 4:** Multivariable logistic regression analysis of risk factors for CMVR.

Variables	Multivariate A			Multivariate B*			Multivariate C**		
	*p* value	Odds Ratio	95%CI	*p* value	Odds Ratio	95%CI	*p* value	Odds Ratio	95%CI
Platelet refractoriness									
No		ref			ref			ref	
Yes	0.0011	5.41	1.98–15.39	0.0006	7.09	2.36–22.86	0.0005	7.78	2.54–25.89
Donor type									
MSD		ref			ref			ref	
HID	0.0034	7.46	2.19–34.87	0.0035	7.32	2.15–34.02	0.0051	6.90	2.00–32.51
URD	0.0030	8.38	2.30–41.34	0.0025	8.61	2.38–41.88	0.0021	9.33	2.52–46.71
Trend	0.0025			0.0019			0.0015		

*adjusted by gender and age; **adjusted by gender, age and diagnosis; CMVR, cytomegalovirus retinitis; MSD, human leukocyte antigen-matched sibling donor; HID, haploidentical donor; URD, unrelated donor.

**Table 5:** The association of pre-transplant factors with post-transplant CMVR risk was analyzed with stratification by gender and age.

Variables	Univariate			Multivariate		
	*p* value	Odds Ratio	95%CI	*p* value	Odds Ratio	95%CI
By gender (female)						
Platelet refractoriness						
No		ref			ref	
Yes	0.0131	5.60	1.47–23.24	0.028	5.02	1.22–22.73
Donor type						
MSD		ref			ref	
HID	0.0434	10.00	1.47–201.28	0.0716	8.27	1.11–172.50
URD	0.0765	7.69	1.08–156.50	0.0881	7.56	0.98–160.04
Trend	0.0772			0.0922		
By gender (male)						
Platelet refractoriness						
No		ref			ref	
Yes	0.0112	6.33	1.51–28.34	0.0119	8.54	1.67–51.72
Donor type						
MSD		ref			ref	
HID	0.0108	7.92	1.92–54.12	0.0169	7.68	1.71–56.56
URD	0.0261	6.63	1.44–47.56	0.0122	9.78	1.93–79.37
Trend	0.0232			0.0090		
By age (< 27 yr)						
Platelet refractoriness						
No		ref			ref	
Yes	0.0027	7.13	1.99–26.71	0.0015	12.64	2.87–72.32
Donor type						
MSD		ref			ref	
HID	0.1440	3.55	0.73–25.91	0.1382	4.16	0.72–35.61
URD	0.0813	4.59	0.93–33.91	0.0881	10.62	1.65–111.72
Trend	0.0798			0.0237		
By age (>= 27 yr)						
Platelet refractoriness						
No		ref			ref	
Yes	0.0404	4.23	1.04–17.43	0.0788	3.57	0.84–15.27
Donor type						
MSD		ref			ref	
HID	0.0046	22.00	3.76–421.67	0.0080	18.59	3.07–360.17
URD	0.0273	12.00	1.82–237.76	0.0352	10.84	1.62–216.07
Trend	0.0188			0.0317		

CMVR, cytomegalovirus retinitis, MSD, human leukocyte antigen-matched sibling donor, HID, haploidentical donor, URD, unrelated donor.

### Model Validation

The prognostic utility of the final model was measured using AUCs. The AUC was 0.65 (95% CI 0.57–0.76), 0.60 (95% CI 0.50–0.76), and 0.62 (95% CI 0.54–0.75) in models A, B, and C, respectively (**Figure 2B**). **Figure 2C** is the nomogram for evaluating the risk of CMVR, with rows ranging from 2 to 4 representing the included variables. The points of the two variables were added to the total points.

## Discussion

In this case-control study of SAA HSCT recipients, two pre-transplant variables—platelet refractoriness and alternative donors—proved to be risk factors for CMVR. This is the first time these two risk factors have been reported in SAA HSCT recipients. Our findings are helpful in guiding clinical decisions about reducing the risk of CMVR and maximizing the benefits of HSCT in SAA patients as part of universal prevention strategies targeting post-transplant CMVR.

CMVR in our study occurred at a median of 148 days after HSCT, which again confirmed that CMVR was more often a late-onset HSCT-related complication, as reported in previous studies (Meng et al., 2020; Yan et al., 2020). The latest onset of CMVR was 386 days after transplantation in our cases, so our non-CMVR controls were observed for at least 2 years to confirm the absence of CMVR. We are not surprised by the finding that alternative donors are associated with an increased risk of developing CMVR, although this is the first report in SAA HSCT recipients. For those without a suitable matched sibling donor, the availability of alternative donors has become the immediate next best option. However, there have been some reports on the particular risk of HSCT-related CMV infection among recipients of alternative grafts (Whited et al., 2020; Cho et al., 2021). We previously also demonstrated the high risk of CMVR associated with HLA-mismatched donors in a retrospective study based on SAA and malignant hematological diseases (Zhang et al., 2017).

The most interesting finding in this study is the predictive value of platelet refractoriness on HSCT-related CMVR. The reasons why patients with pre-transplant platelet refractoriness are more prone to CMVR can be revealed through the following analysis. First, thrombocytopenia is prone to retinal hemorrhage (Kezuka et al., 2005; Wen et al., 2021). In a study of posterior segment complications in HSCT recipients, 90.0% of recipients with retinal hemorrhage had thrombocytopenia (Yoo et al., 2017). Severe anemia and thrombocytopenia coexist in SAA patients. Anemic hypoxic damage to the retinal blood vessel wall, together with low platelet counts, is synergistic factor in the development of retinal bleeding (Ranganath et al., 2011). Second, disruption of the blood–retinal barrier formed by tight junctions of the retinal blood vessel endothelial cells will accelerate CMV spread to the retina, especially in immunosuppressed transplant recipients (Duan et al., 1996; Zhang et al., 2005; Sallam et al., 2018; Xu et al., 2018). Third, the incidence of platelet refractoriness in patients with bone marrow failure can be as high as 50–70% (Murphy and Waters, 1990), which is an independent poor prognosis factor of transplantation (Scott et al., 2016; Fu et al., 2018; Tanoue et al., 2018). SAA is a kind of bone marrow failure, and repeated platelet transfusions can easily result in platelet refractoriness. Before the widespread use of leukoreduction, platelet refractoriness was found in 30% to 70% of patients with bone marrow failure, especially in SAA patients with multiple platelet transfusions (Murphy and Waters, 1990). SAA patients usually face life-threatening bleeding when platelet counts drop below 10 × 10^9^/L, so platelet refractoriness continues to be a challenge for SAA patients, although leukoreduction can lower alloimmune platelet refractoriness from 14% to 4% in patients undergoing chemotherapy or stem cell transplantation (Seftel et al., 2004). Scott A et al. (2018) reported that pre-transplant platelet refractoriness predicts a relatively poor prognosis because these patients with a history of platelet refractoriness were found to enter the intensive care unit earlier for hematologic malignancies post-HSCT. Recently, Yan et al. (2020) revealed an association between platelet engraft failure at 100 days and the development of CMVR in HID HSCT recipients with CMV viremia. These results are consistent with our findings, in which pre-transplant platelet refractoriness is associated with post-transplant CMVR in SAA HSCT recipients. We recommend that the platelet count be closely monitored and thrombopoietin be properly applied during the period when CMVR is prone to occur, mainly aimed at avoiding fundus hemorrhage and reducing the chance of CMV entering the eye through the blood–retinal barrier.

Some limitations of this study must be carefully considered. First, we created this cohort through a retrospective review based on medical records. We selected 13 pre-transplant variables that we assumed had the potential to be associated with the occurrence of CMVR. Most of them are unique features that appear only in SAA, although only a few variables are known risk factors for CMVR reported in malignant hematological diseases. It is possible that some other potential factors were not detected. Another limitation is that we did not confirm the cause and type of platelet refractoriness. We only preliminarily identified pre-transplant platelet refractoriness as a risk factor for CMVR. Further prospective control studies are needed to better predict whether pre-transplant platelet refractoriness could lead to post-transplant CMVR in SAA HSCT recipients.

In conclusion, this is the first study to investigate the association between the pre-transplant status of SAA HSCT recipients and post-transplant CMVR. The most significant finding of this study was that pre-transplant platelet refractoriness and alternative donors can increase the risk of developing CMVR in SAA HSCT recipients. CMVR is a late complication after HSCT, and patients undergoing pre-transplant platelet refractoriness and alternative donors have a higher risk of developing CMVR. We suggest strengthening prevention strategies and fundus screening of post-transplant CMVR for SAA HSCT recipients with a history of pre-transplant platelet refractoriness and alternative donors.

## Data Availability Statement

The original contributions presented in the study are included in the article/supplementary material. Further inquiries can be directed to the corresponding authors.

## Author Contributions

YZ was the major contributor in writing the manuscript. WM and LL designed the research. YL and XZ collected the patient data. JC and LL analysed the data and prepared the tables and figures for the manuscript. YZ, SW, WM, and ZG followed up with patients. All authors reviewed the manuscript.

## Funding

This study was supported by Guangdong Natural Science Foundation (2020A1515010215) and Guangzhou Science and technology planning project (202002030066).

## Conflict of Interest

The authors declare that the research was conducted in the absence of any commercial or financial relationships that could be construed as a potential conflict of interest.

## Publisher’s Note

All claims expressed in this article are solely those of the authors and do not necessarily represent those of their affiliated organizations, or those of the publisher, the editors and the reviewers. Any product that may be evaluated in this article, or claim that may be made by its manufacturer, is not guaranteed or endorsed by the publisher.
